# “Therapeutic Window” in Acute Gonococcal Salpingitis

**DOI:** 10.1155/S1064744995000299

**Published:** 1995

**Authors:** Gilles R. G.

**Affiliations:** Department of Obstetrics and Gynecology Creighton University School of Medicine Omaha Nebraska USA


Infectious Diseases in Obstetrics and Gynecology 3:45–49 (1995)
© 1995 Wiley-Liss, Inc.

### "Therapeutic Window" in Acute Gonococcal Salpingitis

The issue of fertility following acute gonococcal salpingitis has been addressed in less than an optimal manner. The often demographic instability of study patients and the need to publish within specific time frames have partially negated the ability to produce long-term studies of the impact of acute gonococcal salpingitis on subsequent fertility. In his study of acute salpingitis, Heynemann[1] demonstrated that, if antibiotic treatment was started early, before adnexal tumors had formed, the reproductive prognosis for these women was very good. If palpable adnexal masses had developed, the prognosis for fertility was only about 18–20%. Hedberg and Anberg[2] demonstrated that the risk of infertility varied roughly with the duration of disease before treatment. Similarly, Falk[3] demonstrated that the interval between the onset of pain and the initiation of antibiotics was a major factor in prognosticating the ability of such women to become pregnant. The higher the erythrocyte sedimentation rate (ESR) or the larger the adnexal swelling, the poorer was the prognosis for subsequent reproductive outcomes. Hedberg and Spetz[4] reviewed 216 cases of acute salpingitis. Cultures for *Neisseria gonorrhoeae* were positive in 96 and negative in 120 patients. These investigators found a better prognosis for fertility in women who had experienced gonococcal salpingitis compared with those with nongonococcal salpingitis. Viberg[5] surveyed a group of women for voluntary infertility and surgical intervention 2½–5 years after their discharge from the hospital for acute salpingitis. Again, the incidence of pregnancy was higher for patients with gonococcal vs. those with nongonococcal salpingitis. These studies, done in the 1950s and 1960s, correlate well with our current understanding of the pathogenesis of acute gonococcal salpingitis.[6,7] In the absence of a concomitant *Chlamydia trachomatis* infection, gonococcal salpingitis is initially monomicrobial in etiology.[6,8–11] With an alteration of the oxidation-reduction potential, the "anaerobic progression" is initiated.[9–11] The current theory is that anaerobic bacteria are primarily responsible for basement-membrane destruction and healing by fibrosis within the fallopian tube.[9] Early monoetiological gonococcal salpingitis is associated with a relatively limited elevation of ESR (20–45 mm).[6] With a secondary anaerobic bacterial superinfection, levels ⩾60 mm usually indicate the presence of tubal occlusion or a tubo-ovarian complex.

The correlation of cul-de-sac microbiology with clinical response to therapy has demonstrated that, when acute salpingitis was due exclusively to *N. gonorrhoeae*, there was a predictable clinical response: defervescence in 24–36 h, loss of peritoneal signs and most deep organ tenderness in 36–48 h, and a normalization of the WBC count within 24–48 h.[5] When *N. gonorrhoeae* was present as part of a polymicrobial peritonitis or had undergone autoelimination and replacement by an anaerobic isolate, the probability of an altered therapeutic response was greatly enhanced.[6,9–11]

Does a good therapeutic response, thus defined, correlate with reproductive outcomes? Conversely, does an altered therapeutic response correlate in a statistically significant manner with ensuing negative reproductive outcomes? A way to

Received May 30, 1995
Accepted June 5, 1995

Editorial answer this question would be to identify young women who develop gonococcal endocervicitis and salpingitis and meet the following criteria:

1. No prior salpingitis or chlamydial infection
2. A successful pregnancy within a year prior to the occurrence of salpingitis
3. Unprotected coitus with reasonable regularity
4. Availability for follow-up until either pregnancy or a second episode of salpingitis occurs.

Those who meet the anticipated response-to-therapy criteria delineated for monomicrobial salpingitis due to *N. gonorrhoeae* would constitute the control group. Those who respond to therapy differently would constitute the challenge group. The normal control groups for both study populations who had not had salpingitis at any time would be matched for age, race, socioeconomic conditions, locality, and parity. The number of pregnancies per unit of time of the groups would then be statistically compared. Approximately 18 years ago, a study of this nature was attempted. In a period from 1976 through 1981, only 17 cases meeting the study criteria were identified and analyzed. This study was terminated when it became epidemiologically apparent that upper genital tract disease due to *C. trachomatis* might invalidate the observations on even 400 patients. Therefore, the study question (whether there is a period of time between the initial identification of clinical gonococcal salpingitis and effective therapy in which the fallopian-tube structure is relatively preserved) was left unanswered.

Two study patients in the abnormal-response group were unique in that they had sequential culdocenteses and microbiologic characterizations. These patients lend some insight into what is termed the "therapeutic window." These cases are reported not to prove a point but rather to frame the question and encourage others to contribute to the dialogue or challenge the concept proposed.

### CASE REPORTS

### Patient 1

Patient 1 was a 21-year-old, married white female, $P_{1001}$, who presented to the emergency room of the Shands Teaching Hospital with a fever and bilateral lower abdominal pain of approximately 10 h. In the ensuing 14 months in which this woman was sexually active, she had no history of vaginitis or STDs. Four months prior to her admission, she had delivered a 4,111-g boy. A physical examination of the woman revealed rebound tenderness, significant vaginal exudate of the perineum, purulent material coming from the endocervix, and marked cervical motion and adnexal tenderness. A Gram's stain of the endocervix revealed the presence of gram-negative intracellular diplococci. A culdocentesis yielded 3–4 cc of pus, and a Gram's stain revealed the presence of rare gram-negative intracellular diplococci.

Having been judged to have monoetiological gonococcal salpingitis, the patient was started on minocycline therapy. She had an initial lysis temperature (Fig. 1) which was followed approximately 36 h after the initial culdocentesis by a progressive elevation of temperature. At the time of a repeat culdocentesis, her temperature (39°C) had exceeded her highest initial temperature (38.6°C). A Gram's stain of the cul-de-sac aspirate yielded the presence of small gram-positive cocci. The patient was started on ampicillin to which she exhibited an excellent therapeutic response.

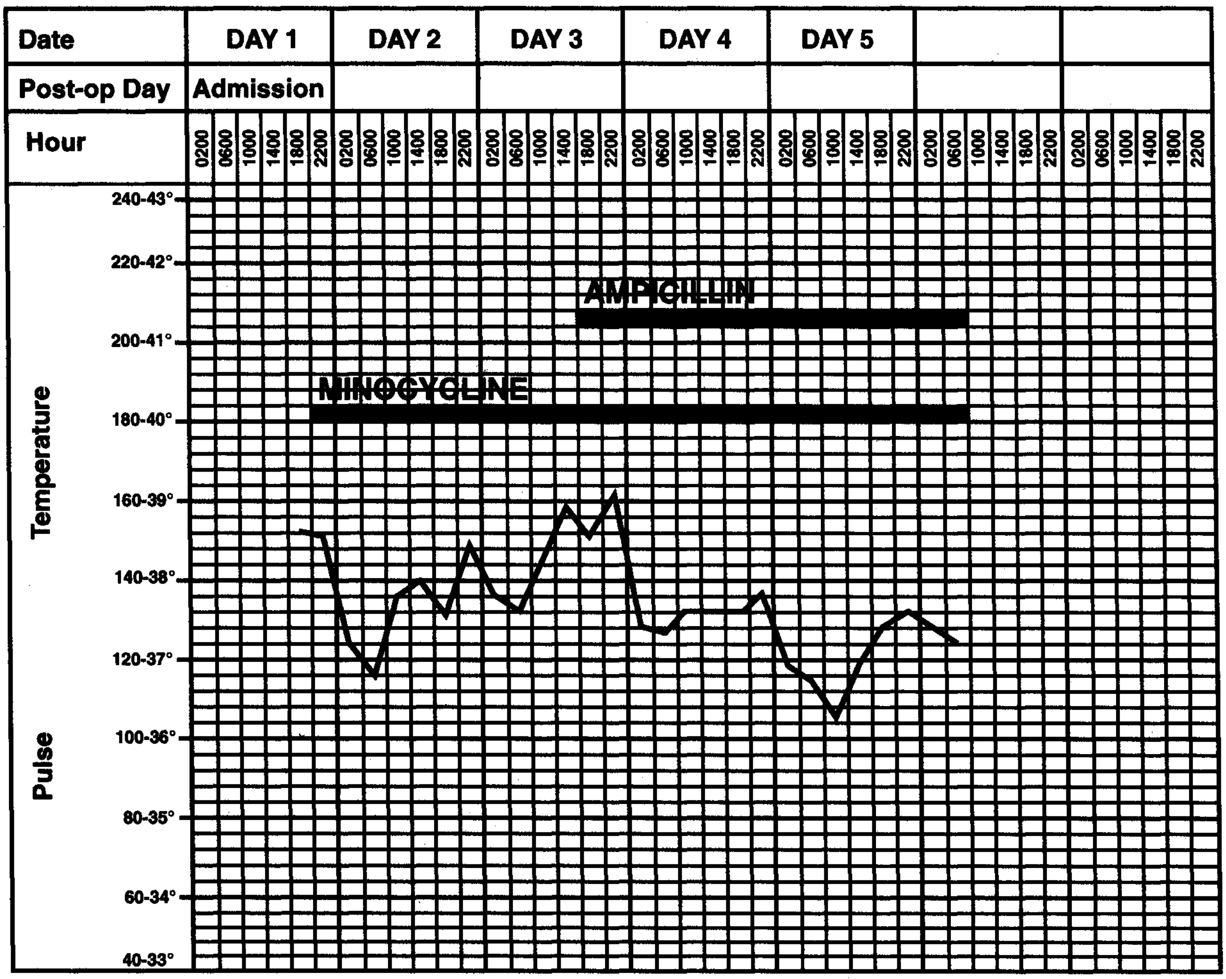

Fig. 1. Temperature chart of a patient with gonococcal endocervicitis and gonococcal polymicrobial peritonitis. After the initial lysis of fever, the patient had a prolonged temperature elevation due to superinfecting *Peptostreptococcus.*

The endocervical cultures grew out *N. gonorrhoeae. N. gonorrhoeae,* a *Bacteroides* species that was sensitive to tetracycline but resistant to penicillin, and a *Peptostreptococcus* that was sensitive to penicillin but resistant to tetracycline were isolated from the cul-de-sac.

For the 27 months following her discharge in which she was monitored, she and her husband practiced unprotected coitus without achieving a conception.

### Patient 2

Patient 2 was a 28-year-old, multigravid, $P_{4004}$, black female who was brought in from a rural community because of the presence of severe lower abdominal pelvic pain and fever for 12 h. In the emergency room, the patient was found to have a temperature of 39°C and significant deep organ tenderness. No significant rebound tenderness was identified. A pelvic examination revealed the presence of a clinically significant vaginal discharge on the perineum and at the endocervix and marked cervical motion and adnexal tenderness. A Gram's stain from the endocervix revealed gram-negative intracellular diplococci. *N. gonorrhoeae* was recovered from the endocervical swab. A wet mount revealed the presence of *T. vaginalis.* A

culdocentesis yielded a minimum amount of slightly turbid fluid. No bacteria were subsequently recovered. Before therapy could be instituted, the patient left the emergency room. She was transported back to the emergency room approximately 27 h later. At that time, marked rebound tenderness was present. A repeat culdocentesis yielded an unspecified amount of pus from which *Gardnerella vaginalis, Peptostreptococcus,* and *Enterococcus* were isolated. The patient was placed on aggressive triple antibiotic therapy and was discharged 4 days later on doxycycline.

This woman's last child had been delivered 7 months previously. She was subsequently monitored for a period of 17 months during which time she and her husband were practicing unprotected coitus without the establishment of a pregnancy.

### DISCUSSION

The ability to assess the interval in which polymicrobial endosalpingitis must exist before fallopian-tube function is effectively compromised is pragmatically and ethically limited. Having sequential culdocentesis specimens that document the prior progression of gonococcal salpingitis, the birth of a baby 7 months previously, biological confirmation of both tubal patency and no male factor, and long-term follow-up afford some insight into the problem. Both reported cases fulfilled the criteria set forth for therapeutic success by the CDC: eradication of causative agent, nonprogression of disease to a tubo-ovarian complex, and discharge from the hospital in ≤5 days; but both cases failed to fulfill the Gainesville criteria of the anticipated therapeutic response described for monoetiological disease. The persistence of polymicrobial disease for 24–36 h in these 2 cases precluded the subsequent ability of effective therapy to achieve the primary therapeutic goal for acute salpingitis, that is, the preservation of fallopian-tube function.

In individual cases the therapeutic window appears to be limited to as little time as 25 h.[9–11] If prior structural damage to the fallopian tubes existed as a consequence of previous gonococcal or chlamydial disease, one can hypothesize that the therapeutic window might be shorter than projected. These 2 cases are presented in the hope of stimulating research to further our understanding of the natural history of permanent fallopian tube damage following acute salpingitis and help define the interval of the therapeutic window in which appropriate antibiotic intervention may prevent the sequelae leading to tubal damage.

*Gilles R.G. Monif*
*Department of Obstetrics and Gynecology*
*Creighton University School of Medicine*
*Omaha, Nebraska*

### REFERENCES

1. Heynemann T: Entzüdung der Adnexe. In Seitzl, Amrelch AI (eds): Biologix unde Pathologic des Weibes, 5. band. Berlin: Urban und Schwarzenberg, p 19, 1953.
2. Hedberg E, Anberg A: Gonorrheal salpingitis: Views on treatment and progress. Fertil Steril 16:125–133, 1965.
3. Falk V: Treatment of acute non-tuberculous salpingitis with antibiotics alone and in combination with glucocorticoids with special reference to laparoscopy. Acta Obstet Gynaecol Scand 44 (Suppl 6):1–118, 1965.
4. Hedberg E, Spetz, SO: Acute salpingitis: Views on prognosis and treatment. Acta Obstet Gynaecol Scand 37:131–148, 1958.
5. Viberg L: Diagnosis of acute salpingitis: Interpretation of the pathogens with special reference to laparoscopy. Acta Obstet Gynaecol Scand 54 (Suppl 6):1965.

6. Monif GRG: Acute Salpingitis in Infectious Diseases in Obstetrics and Gynecology. 3rd ed. Omaha, NE: IDI Publications, pp 4–79–4–99, 1993.
7. Cunningham FG, Hauth JC, Gilstrap LC, et al.: The bacterial pathogenesis of acute pelvic inflammatory disease. Obstet Gynecol 52:161, 1978.
8. Westrom L: Effect of acute pelvic inflammatory disease on fertility. Am J Obstet Gynecol 121:707–713, 1975.
9. Monif GRG: Choice of antibiotics and length of therapy in the treatment of acute salpingitis. Am J Med 78:188–193, 1985.
10. Monif GRG, Welkos SL, Baer H, Thompson RJ: Cul-de-sac isolates from patients with endometritis/salpingitis/peritonitis and gonococcal endocervicitis. Am J Obstet Gynecol 126: 158–161, 1976.
11. Monif GRG, Welkos SL, Baer H: Clinical response of patient with gonococcal endocervicitis and endometritis-salpingitis-peritonitis to doxycycline. Am J Obstet Gynecol 129:614–622, 1977.
